# Supplementary material for: Burden of Disease (BoD) Assessment to Estimate Risk Factors Impact in a Real Nanomanufacturing Scenario
Source: Nanomaterials (Basel). 2022 Nov 21;12(22):4089. doi: 10.3390/nano12224089 (PMC9696424; doi:10.3390/nano12224089)
Supplement: Supplementary file 1 [file nanomaterials-12-04089-s001.zip › nanomaterials-1987874-supplementary/nanomaterials-1987874-supplementary 11.20 for conversion part 1.pdf]

SUPPLEMENTARY MATERIAL

Burden of Disease (BoD) assessment to estimate risk factors impact in a real nanomanufacturing scenario

Antti Joonas Koivisto [1,2,3,*], Marko Altin [4], Irini Furxhi [5,6], Maxime Eliat [3], Sara Trabucco [7], Magda Blosi [8],
Jesús Lopez de Ipiña [9], Franco Belosi [7] and Anna Costa [8]

[1] Air Pollution Management APM, Mattilanmäki 38, 33610 Tampere, Finland
[2] Institute for Atmospheric and Earth System Research (INAR), University of Helsinki, PL 64, 00014 Helsinki, Finland
[3] ARCHE Consulting, Liefkensstraat 35D, 9032 Wondelgem, Belgium
[4] Witek s.r.l., Via Siena 47, 50142 Firenze, Italy
[5] Transgero Limited, Cullinagh, Newcastle West, Co. Limerick, V42 V384 Limerick, Ireland
[6] Department of Accounting and Finance, Kemmy Business School, University of Limerick, V94 T9PX Limerick, Ireland
[7] CNR-ISAC, Institute of Atmospheric Sciences and Climate, National Research Council of Italy, Via Gobetti, 101, 40129 Bologna, Italy
[8] ISTEC-CNR, Institute of Science and Technology for Ceramics, CNR, National Research Council, Via Granarolo 64, 48018 Faenza, Italy
[9] TECNALIA, Basque Research and Technology Alliance (BRTA), Parque Tecnológico de Alava, Leonardo Da Vinci 11, 01510 Miñano, Spain
* Correspondence: joonas.apm@gmail.com; Tel.: +358-407-222-029

Field measurement of the Local Exhaust Ventilation (LEV) M5 filter filtration efficiency

According to EN 779:2012 and EN 1822:2019 M5 type filter filtration efficiency for 0.4 µm particles is between 40% and 60%. The filtration efficiency may vary depending on the filter loading and particle type. Previous studies investigating Witek spray process emissions did not report the measured M5 filter filtration efficiency even though measurements were carried out before and after the LEV filter (Del Secco et al., 2022; Koivisto et al., 2022).

Measurements

Particle number concentrations were measured in the spray chamber and at the LEV duct after the M5 filter as described by Del Secco et al. (2022) by using two low-cost optical particles counters (SPS30, Sensirion, Staefa, Switzerland). SPS30 measure particle number concentration with diameter over 0.3 µm in the particle count range 0–3000 $1/cm^3$ and classifies the particles according to their optical size in four classes: 0.5–1 µm, 1.0–2.5 µm, 2.5–4 µm, and 4–10 µm. Measurements were carried out over all tests 1 to 12 as described by Del Secco et al. (2022) and Koivisto et al. (2022). Tests 1 to 6 refer to TiO_2N and 7 to 12 AgHEC spray experiments.

Results

The spray chamber total concentrations varied from 4823 to 15183 $1/cm^3$ in TiO_2N experiments (tests 1 to 6) and from 607 to 1919 $1/cm^3$ in AgHEC experiments (test 7 to 12). The LEV concentration after M5 filter varied from 2784 to 9593 $1/cm^3$ in TiO_2N experiments and from 272 to 723 $1/cm^3$ in AgHEC experiments. Table S1 shows the average filtration efficiency calculated from TiO_2N experiments 1 to 6 and AgHEC experiments 7 to 12. The spray chamber concentration exceeded the upper count limit in TiO_2N and the results can only provide lower limit for the filtration efficiency. AgHEC filtration efficiency shows that the filtration performance in this setting was 60 ± 13 % which is in agreement with the M5 filter specifications.

Table S1. Average filtration efficiency and standard deviation calculated from TiO_2N and AgHEC experiments.

**Coating process**	**Filtration efficiency**		
	**0.3-0.5 µm**	**0.5-1.0 µm**	**1-10 µm**
TiO_2N	>23 ± 8	>34 ± 8	>39 ± 10
AgHEC	60 ± 13	64 ± 14	68 ± 14

References

Del Secco, B., Trabucco, S., Ravegnani, F., Koivisto, A.J., Zanoni, I., Blosi, M., Ortelli, S., Altin, M., Bartolini, G., Costa, A.L., Belosi, F., 2022. Particles Emission from an Industrial Spray Coating Process Using Nano-Materials. Nanomaterials 12, 313. https://doi.org/10.3390/nano12030313

EN 779:2012. Particulate air filters for general ventilation - Determination of the filtration performance.

EN 1822, 2019. High efficiency air filters (EPA, HEPA and ULPA) - Part 1: Classification, performance testing, marking.

Koivisto, A.J., Del Secco, B., Trabucco, S., Nicosia, A., Ravegnani, F., Altin, M., Cabellos, J., Furxhi, I., Blosi, M., Costa, A., Lopez de Ipiña, J., Belosi, F., 2022. Quantifying Emission Factors and Setting Conditions of Use According to ECHA Chapter R.14 for a Spray Process Designed for Nanocoatings—A Case Study. Nanomaterials 12, 596. https://doi.org/10.3390/nano12040596
