# Supplementary material for: Burden of Disease (BoD) Assessment to Estimate Risk Factors Impact in a Real Nanomanufacturing Scenario
Source: Nanomaterials (Basel). 2022 Nov 21;12(22):4089. doi: 10.3390/nano12224089 (PMC9696424; doi:10.3390/nano12224089)
Supplement: Supplementary file 1 [file nanomaterials-12-04089-s001.zip › nanomaterials-1987874-supplementary/nanomaterials-1987874-supplementary 11.20 for conversion part 2.pdf]

	Naam	Omschrijving
**PROJECT**	Witek TiO2	Emission impact assessment for Witek coating process
**SCENARIO**	Coating with single machine	

Parametrisatie

Naam Polluent	Droge depositiesnelheid (m/s)	Uitwascoëfficiënt λ (s-1)	Molaire massa (g/mol)	% stikstof	Zuurequivalent (Zeq/mol)
TIO2	0	4.0E-4			

**Receptorhoogte (m)**	1,5	**Gebruikt meteobestand**	2007-2011
**Percentielen berekenen**	Nee	**Achtergrondconcentratiekaart**	Geen
**Stack downwash gebruiken**	Ja	**Koppeling VLOPS-depositiesnelhedenkaart**	UIT

Bronkarakteristieken

Naam	Type
LEV stack	Puntbron
Fugitive point source	Puntbron

Bron LEV stack

**Naam**	LEV stack	**Hoogte (m)**	13
**NO/NOx verhouding (%)**		**Werkingsregime**	variabel
**Temperatuurafhankelijk**	Nee	**Uittreedsnelheid (m/s)**	3,11
**Windafhankelijk**	Nee		
**Diameter (m)**	0,6	**Temperatuur (K)**	296,15
**Volumestroom (Nm^3/s)**	0,81	**Mechanische pluimstijging uitschakelen**	Nee
**Locatie**		**X**	100000
		**Y**	200000

Emissies LEV stack

Polluent	Waarde	Massaeenheid	Tijdseenheid
TIO2	149,82	mg	minuut

Effectieve pluimhoogte LEV stack

	Windsnelheidsklasse (m/s op 24 m)														
**Stab. klasse**	**1**	**2**	**3**	**4**	**5**	**6**	**7**	**8**	**9**	**10**	**11**	**12**	**13**	**14**	**15**
**E1**	19	17	15	14	13	13	12	12	12	12	11	11	11	11	11
**E2**	19	17	15	14	13	13	12	12	12	12	11	11	11	11	11
**E3**	20	16	15	14	13	12	12	12	12	11	11	11	11	11	11
**E4**	19	16	15	13	13	12	12	12	12	11	11	11	11	11	11
**E5**	19	16	14	13	13	12	12	12	11	11	11	11	11	11	11
**E6**	19	16	14	13	13	12	12	12	11	11	11	11	11	11	11
**E7**	20	16	15	14	13	12	12	12	12	11	11	11	11	11	11

Bron Fugitive point source

**Naam**	Fugitive point source	**Hoogte (m)**	3
**NO/NOx verhouding (%)**		**Werkingsregime**	variabel
**Temperatuurafhankelijk**	Nee	**Uittreedsnelheid (m/s)**	0,02
**Windafhankelijk**	Nee		
**Diameter (m)**	10	**Temperatuur (K)**	296,15
**Volumestroom (Nm^3/s)**	1,5	**Mechanische pluimstijging uitschakelen**	Ja
**Locatie**		**X**	100001,13
		**Y**	199998,63

Emissies Fugitive point source

Polluent	Waarde	Massaeenheid	Tijdseenheid
TIO2	15,1	mg	minuut

Effectieve pluimhoogte Fugitive point source

	Windsnelheidsklasse (m/s op 24 m)														
**Stab. klasse**	**1**	**2**	**3**	**4**	**5**	**6**	**7**	**8**	**9**	**10**	**11**	**12**	**13**	**14**	**15**
**E1**	7	3	2	2	1	1	1	1	1	1	1	1	1	0	0
**E2**	7	3	2	2	1	1	1	1	1	1	1	1	1	0	0
**E3**	7	3	2	2	1	1	1	1	1	1	1	1	1	0	0
**E4**	9	5	3	2	2	2	1	1	1	1	1	1	1	1	1
**E5**	9	4	3	2	2	1	1	1	1	1	1	1	1	1	1
**E6**	8	4	3	2	2	1	1	1	1	1	1	1	1	1	1
**E7**	7	3	2	2	1	1	1	1	1	1	1	1	1	0	0

Receptoren

Type	Aantal receptoren
Receptor punten	0
Grids	400
Totaal	400

Berekening IMPACT${build.version}

Id	Berekend door	Einde berekening	Invoerbestand	Uitvoerbestand
44651914	Antti Jonas Koivisto	11/05/2022 10:54:15	ifdmjob-44651914-input.zip	ifdmjob-44651914-output.zip

Visualisaties

Id	Laatst gewijzigd op	Laatst gewijzigd door	Polluent	Grootheid	Metriek	Eenheid
44303248	29/04/2022 09:48:55	Antti Jonas Koivisto	TIO2	Concentratie	Gemiddelde	ng/m³

TIO2 (Concentratie Gemiddelde ng/m³)

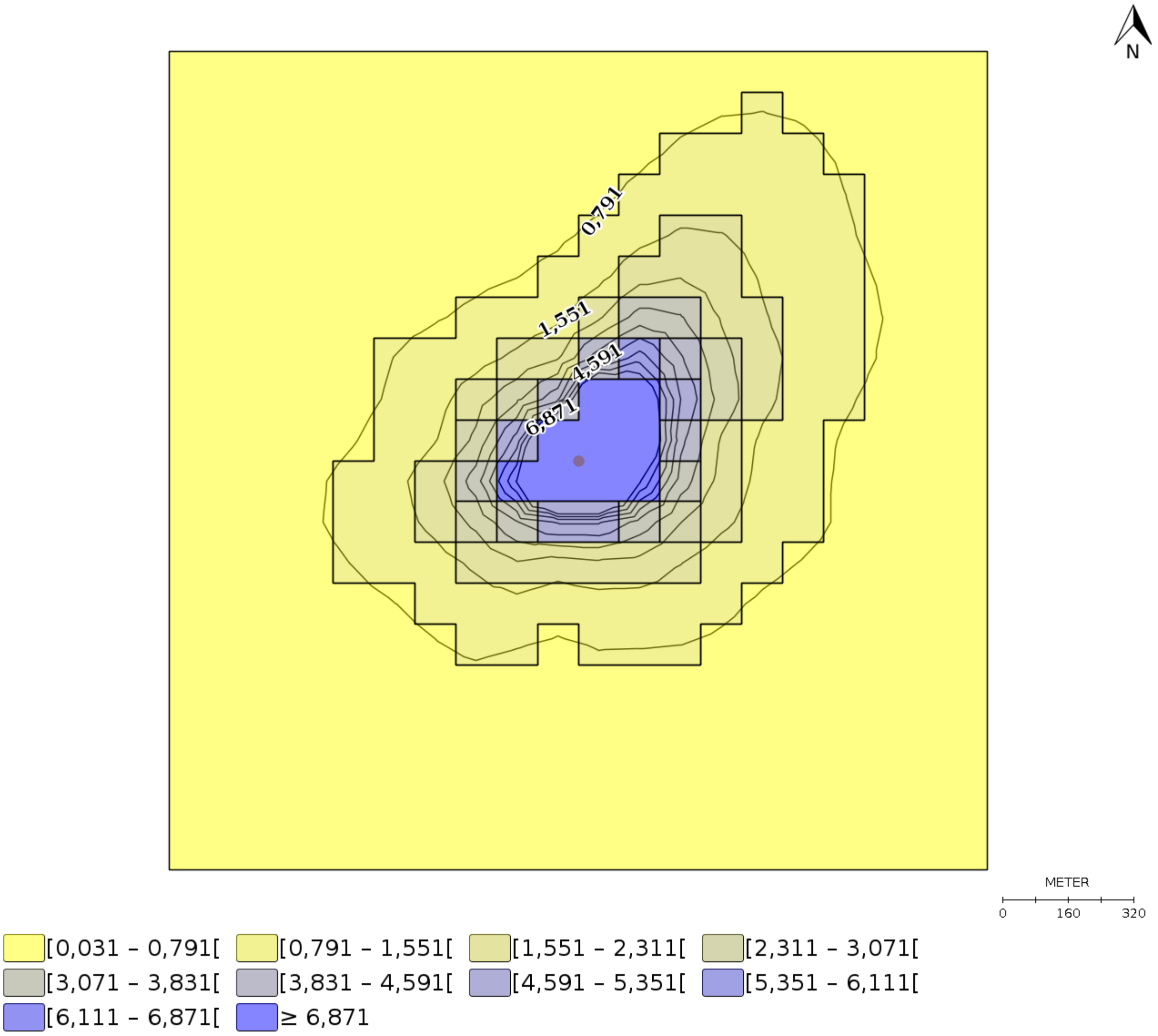

Id	Laatst gewijzigd op	Laatst gewijzigd door	Polluent	Grootheid	Metriek	Eenheid
44303259	11/05/2022 10:45:51	Antti Jonas Koivisto	TIO2	Depositie	Gemiddelde	µg/m²/dag

TIO2 (Depositie Gemiddelde µg/m²/dag)

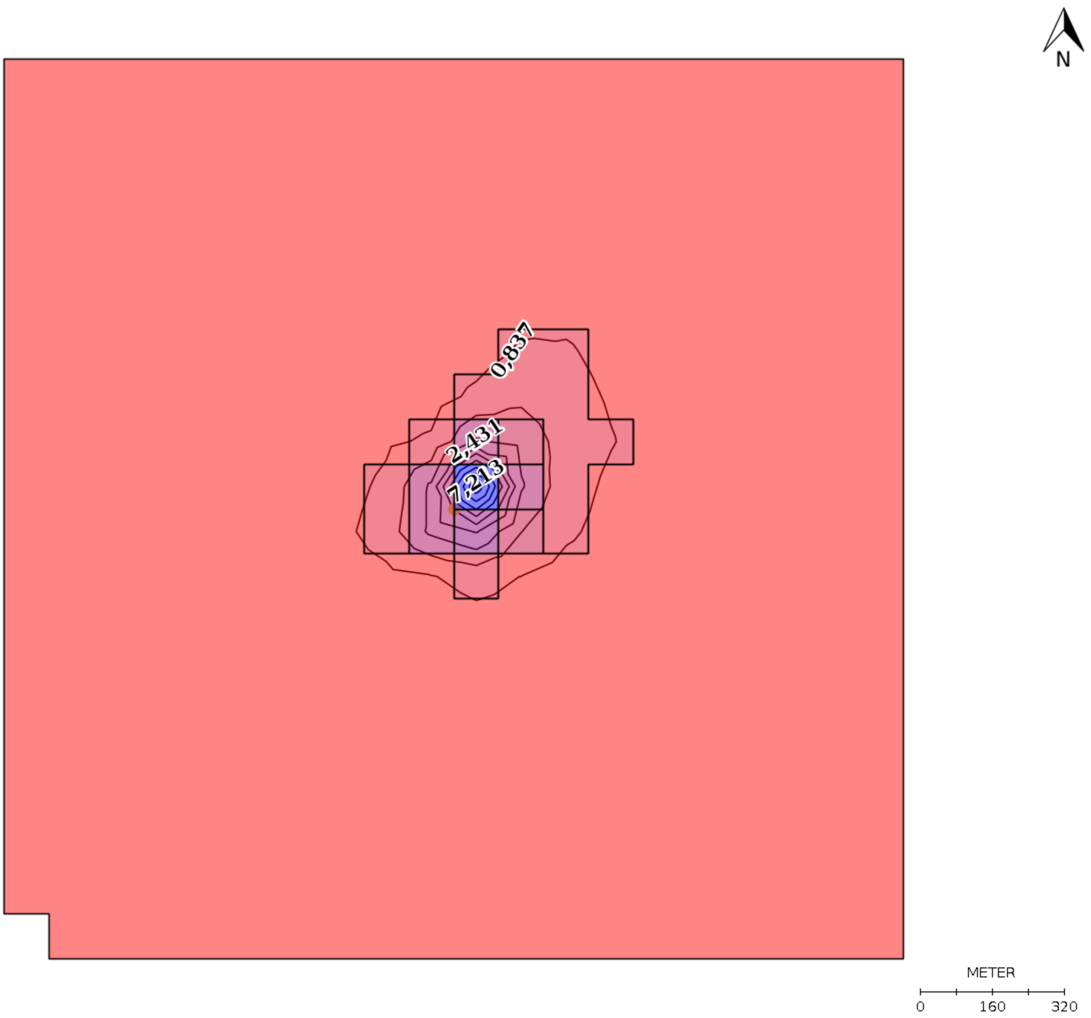

[0,040 – 0,837[| [0,837 – 1,634[| [1,634 – 2,431[| [2,431 – 3,228[
[3,228 – 4,025[| [4,025 – 4,822[| [4,822 – 5,619[| [5,619 – 6,416[
[6,416 – 7,213[| ≥ 7,213

	Naam	Omschrijving
**PROJECT**	Witek Ag	Emission impact assessment for Witek coating process
**SCENARIO**	Coating with single machine	

Parametrisatie

Naam Polluent	Droge depositiesnelheid (m/s)	Uitwascoëfficiënt λ (s-1)	Molaire massa (g/mol)	% stikstof	Zuurequivalent (Zeq/mol)
SILVER	0	4.0E-4			

**Receptorhoogte (m)**	1,5	**Gebruikt meteobestand**	2007-2011
**Percentielen berekenen**	Nee	**Achtergrondconcentratiekaart**	Geen
**Stack downwash gebruiken**	Ja	**Koppeling VLOPS-depositiesnelhedenkaart**	UIT

Bronkarakteristieken

Naam	Type
LEV stack	Puntbron
Fugitive point source	Puntbron

Bron LEV stack			
**Naam**	LEV stack	**Hoogte (m)**	13
**NO/NOx verhouding (%)**		**Werkingsregime**	variabel
**Temperatuurafhankelijk**	Nee	**Uittreedsnelheid (m/s)**	3,11
**Windafhankelijk**	Nee		
**Diameter (m)**	0,6	**Temperatuur (K)**	296,15
**Volumestroom (Nm^3/s)**	0,81	**Mechanische pluimstijging uitschakelen**	Nee
**Locatie**		**X**	100000
		**Y**	200000

Emissies LEV stack			
**Polluent**	**Waarde**	**Massaeenheid**	**Tijdseenheid**
SILVER	2,8	mg	minuut

Effectieve pluimhoogte LEV stack															
	Windsnelheidsklasse (m/s op 24 m)														
**Stab. klasse**	**1**	**2**	**3**	**4**	**5**	**6**	**7**	**8**	**9**	**10**	**11**	**12**	**13**	**14**	**15**
**E1**	19	17	15	14	13	13	12	12	12	12	11	11	11	11	11
**E2**	19	17	15	14	13	13	12	12	12	12	11	11	11	11	11
**E3**	20	16	15	14	13	12	12	12	12	11	11	11	11	11	11
**E4**	19	16	15	13	13	12	12	12	12	11	11	11	11	11	11
**E5**	19	16	14	13	13	12	12	12	11	11	11	11	11	11	11
**E6**	19	16	14	13	13	12	12	12	11	11	11	11	11	11	11
**E7**	20	16	15	14	13	12	12	12	12	11	11	11	11	11	11

Bron Fugitive point source			
**Naam**	Fugitive point source	**Hoogte (m)**	3
**NO/NOx verhouding (%)**		**Werkingsregime**	variabel
**Temperatuurafhankelijk**	Nee	**Uittreedsnelheid (m/s)**	0,02
**Windafhankelijk**	Nee		
**Diameter (m)**	10	**Temperatuur (K)**	296,15
**Volumestroom (Nm^3/s)**	1,5	**Mechanische pluimstijging uitschakelen**	Ja
**Locatie**		**X**	100001,13
		**Y**	199998,63

Emissies Fugitive point source			
**Polluent**	**Waarde**	**Massaeenheid**	**Tijdseenheid**
SILVER	23,2	µg	minuut

Effectieve pluimhoogte Fugitive point source															
	Windsnelheidsklasse (m/s op 24 m)														
**Stab. klasse**	**1**	**2**	**3**	**4**	**5**	**6**	**7**	**8**	**9**	**10**	**11**	**12**	**13**	**14**	**15**
**E1**	7	3	2	2	1	1	1	1	1	1	1	1	1	0	0
**E2**	7	3	2	2	1	1	1	1	1	1	1	1	1	0	0
**E3**	7	3	2	2	1	1	1	1	1	1	1	1	1	0	0
**E4**	9	5	3	2	2	2	1	1	1	1	1	1	1	1	1
**E5**	9	4	3	2	2	1	1	1	1	1	1	1	1	1	1
**E6**	8	4	3	2	2	1	1	1	1	1	1	1	1	1	1
**E7**	7	3	2	2	1	1	1	1	1	1	1	1	1	0	0

Receptoren

Type	Aantal receptoren
Receptor punten	0
Grids	400
Totaal	400

Berekening IMPACT${build.version}

Id	Berekend door	Einde berekening	Invoerbestand	Uitvoerbestand
44653745	Antti Jonas Koivisto	11/05/2022 12:52:56	ifdmjob-44653745-input.zip	ifdmjob-44653745-output.zip

Visualisaties

Id	Laatst gewijzigd op	Laatst gewijzigd door	Polluent	Grootheid	Metriek	Eenheid
44653722	11/05/2022 12:53:50	Antti Jonas Koivisto	SILVER	Concentratie	Gemiddelde	pg/m³

SILVER (Concentratie Gemiddelde pg/m³)

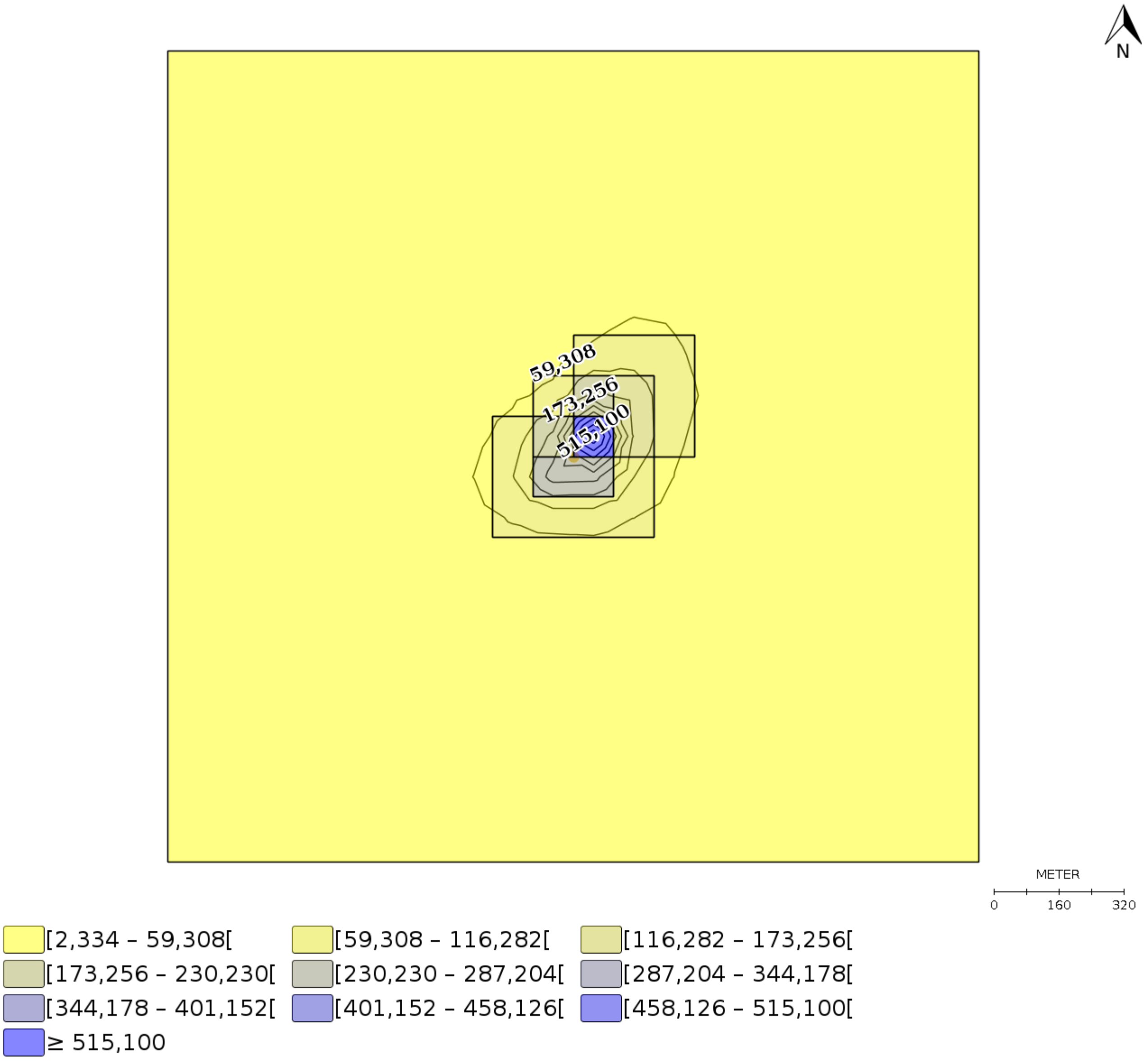

Id	Laatst gewijzigd op	Laatst gewijzigd door	Polluent	Grootheid	Metriek	Eenheid
44653733	11/05/2022 12:57:31	Antti Jonas Koivisto	SILVER	Depositie	Gemiddelde	µg/m²/dag

SILVER (Depositie Gemiddelde µg/m²/dag)

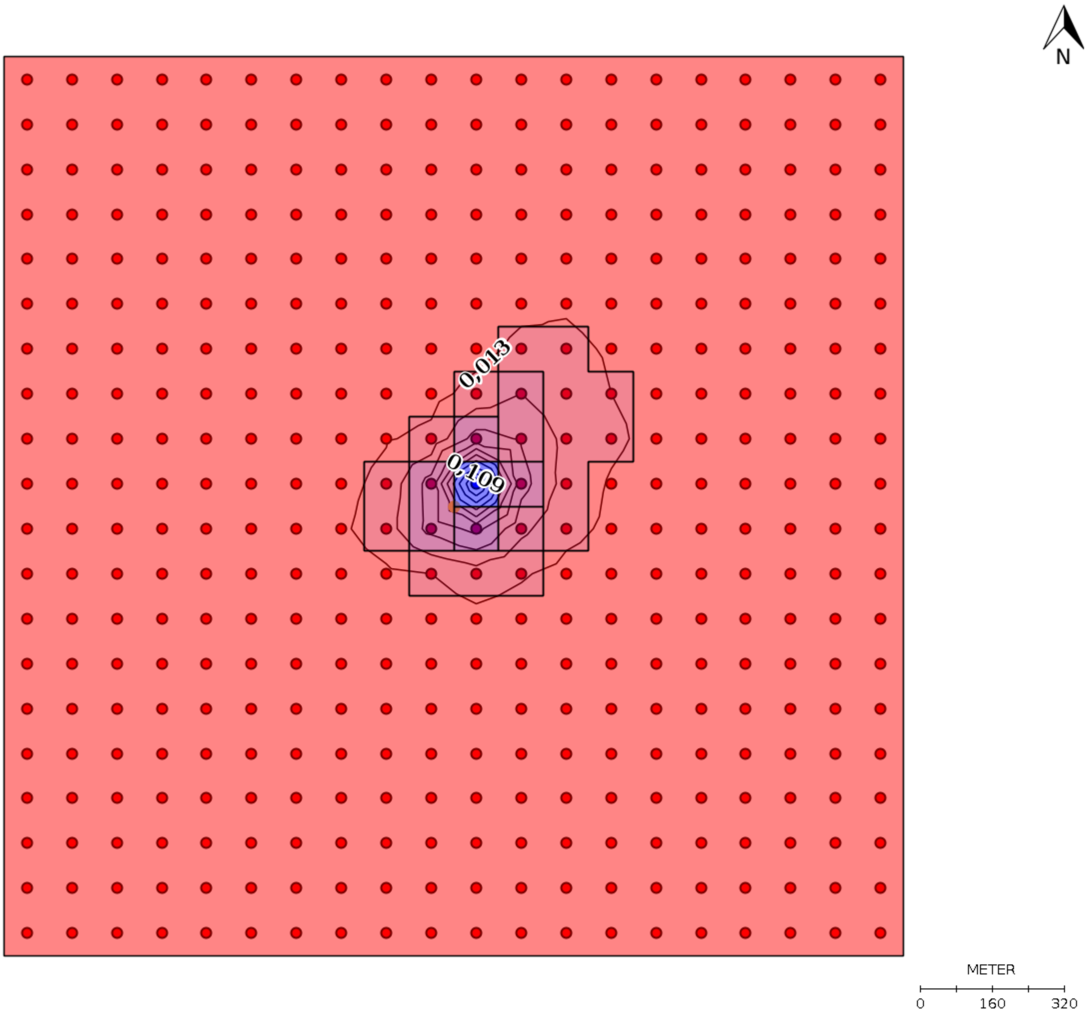

[0,001 – 0,013[
[0,013 – 0,025[
[0,025 – 0,037[
[0,037 – 0,049[
[0,049 – 0,061[
[0,061 – 0,073[
[0,073 – 0,085[
[0,085 – 0,097[
[0,097 – 0,109[
≥ 0,109
